# Women Dentists in Education, Specialization, and Leadership: A Global Survey

**DOI:** 10.1016/j.identj.2026.109522

**Published:** 2026-03-27

**Authors:** Amely Hartmann, Martina Elisabeth Werner, Juliane von Hoyningen-Huene, Guglielmo Campus, Simona Dianišková, Thomas Gerhard Wolf, FDI Section Women Dentists Worldwide Collaboration Group

**Affiliations:** a Clinic for Oral Surgery and Implantology Dr. Seiler und Kollegen MVZ, Filderstadt, Germany; b Department of Oral and Maxillofacial Surgery, Plastic Surgery, University Medical Centre of the Johannes Gutenberg University of Mainz, Mainz, Germany; c Department of Restorative, Preventive and Pediatric Dentistry, School of Dental Medicine, University of Bern, Bern, Switzerland; d Private Practice, Berlin, Germany; e Department of Cariology, Institute of Odontology, Sahlgrenska Academy, University of Gothenburg, Gothenburg, Sweden; f Department of Dental and Maxillo-Facial Sciences, La Sapienza University of Rome, Rome, Italy; g Department of Orthodontics, Medical Faculty, Slovak Medical University, Bratislava, Slovakia; h Department of Periodontology and Operative Dentistry, University Medical Center of the Johannes Gutenberg University Mainz, Mainz, Germany

**Keywords:** Female dentists, Leadership, Women dentists

## Abstract

**Introduction and aims:**

This study aimed to collect data of national dental associations (NDAs) on workforce composition, employment patterns, academic representation, specialization, and women’s participation in leadership roles.

**Methods:**

A validated online-questionnaire was distributed to 189 NDAs across 133 countries within the FDI World Dental Federation-section Women-Dentists-Worldwide. Responses were categorized into five geo-areas. χ² tests and multivariate multinomial logistic regression analyses were used to examine the independent correlations between sex distribution, specialization, pay disparities, and career progression.

**Results:**

Forty-four NDAs from 37 countries participated (23.3%). In 80% of NDAs, women represented >50% of dental students; in 44.5% enrolment exceeded 60%. Female specialization rates varied by region (Europe 24.6%, Central/North America 19.1%, Asia 15.3%), while women remained underrepresented in surgical fields (1.5%-2.9%). Leadership representation was limited: 34.2% of NDAs reported 0% to 20% female professors and 24.4% reported 21% to 40%. Female deans were reported in 41.5% (0%-20%), 19.5% (21%-40%), and 12.2% (81%-100%) of NDAs. In scientific associations, 43.2% reported 0% to 20% female presidents, 11.4% reported 81% to 100%. Council representation clustered at 0-20% (22.7%) and 21-40% (31.8%); assemblies at 13.6% (0%-20%), 29.5% (21%-40%) and 22.7% (41%-60%). Regarding gender-based pay, 67.6% of NDAs reported no differences, 29.7% report no data, only one NDA reported a 35% pay gap in private sector.

**Conclusions:**

Despite high female student representation, women remain underrepresented in academic and professional leadership. Reported barriers included uneven family responsibilities and limited mentoring. These findings highlight the need for structural measures that support equitable career development and inclusive policies in dentistry.

**Clinical relevance:**

Although women increasingly study dentistry worldwide, they remain underrepresented in specializations and leadership positions, potentially affecting workforce composition, academic career, and organizational structures. Structural measures are essential to promote equitable career development and inclusive policies within the dental profession.

## Introduction

Women constitute approximately 41.2% of the global workforce yet hold only 28.8% of leadership/senior positions.^1^ In 2022, women accounted for 37.5% of leadership hires; however, this figure declined to 36.4% by 2024.^2^ In the education sector, women represented only 30% of leadership positions.^3^ In dentistry, the representation of women has increased over the past five decades.^4^ In the 19th century, female students comprised merely 2% of first-year cohorts. By the mid-1980s, this proportion rose to 19.8%, and by 1990 to 38%. Today, sex distribution among dental students is nearly balanced, with women composing approximately half of all first-year enrollments.^5^^,^^6^ Despite this progress, sex disparities remain evident.^6^ Women in dentistry exhibit lower rates of postgraduate qualification attainment and remain underrepresented in leadership roles across scientific, academic, and political spheres.^7^ A discernible decline in female representation is visible as one ascends the academic hierarchy.^8^, ^9^, ^10^, ^11^, ^12^, ^13^ A persistent gender pay gap further compounds this inequality. In 2023, the OECD (Organization for Economic Cooperation and Development) median earnings disparity between male and female full-time employees stood at 11.3% of male median earnings. Across most professions – including medicine and dentistry^14^^,^^15^ – women earn 20% to 30% less for equivalent roles.^16^^,^^17^ For instance, a global analysis on dentists reports female full-time self-employed practitioners earning 37% less than their male counterparts.^10^ Within academic dentistry, women remain underrepresented in senior academic ranks.^11^^,^^13^^,^^15^ In the US, data from 2011 to 2023 show women making up ∼36.2% of faculty, with steady increases yet pronounced underrepresentation in senior roles; full professors and associate professors saw increases of 23.9% and 4.7%, respectively.^5^ Another study covering 2011 to 2019 indicates women's share in instructor roles at 56% to 65%, but as low as 18% to 26% in professorships.^7^ A 2023 survey found that 76% of women faculty held leadership roles – up from 53% in 2015 – but 71% still perceived compensation inequities.^8^ Intersectional data reveal that in 2018 to 2019, women of colour comprised only 7.3% of dental school deans, compared to 17.6% for white women.^9^ Entrepreneurial sectors also mirror these disparities. Women-led startups receive significantly less investment capital, yet generate 10% more revenue over five years.^18^ There is a notable lack of women-led startup teams obtaining funding from venture capitalists and fewer women-led teams securing patents.^19^ The Global Gender Gap Report 2025 shows that, at current progress rates, achieving full gender parity globally will take 123 years.^1^ Therefore, this global survey aims to evaluate gender equality in dentistry by looking at how many women are in training, specialization, and leadership positions. It seeks to identify structural gaps and examine how the large number of female students and the lower rate of specialization and leadership could affect the composition of workforce.

## Material and methods

### Questionnaire development

The preliminary version of the questionnaire was designed based on three primary sources: a comprehensive review of the relevant literature, the collection of available global data, and the experiential insights of the research team and expert group. The pilot version was independently reviewed and refined by a working group. This working group comprised researchers from the University of Bern (Switzerland) and a group of female dentists from the FDI-Section Women Dentists Worldwide (WDW) of the FDI World Dental Federation.^20^ The questionnaire design of this structured, closed cross-sectional survey was based on the modified Delphi method and Stehr-Green scale.^21^ The English-language online questionnaire, including an information sheet on study objectives, was distributed to national dental associations contact persons via an individual link sent by e-mail. Participants were required to accept informed consent before accessing the questionnaire. The validated questionnaire (n = 15) comprised 56 items on a single screen page. All items showed high reliability (ICC > 0.80) in the pretest. The questionnaire could be completed on various devices (PC, laptop, smartphone). This study adhered to the CHERRIES guidelines.^22^ The survey collected basic information about the respective country and the participating national dental association, recorded demographic information about the dental profession, and gathered data on sex-specific aspects of professional and academic development. Overall, the questionnaire provided an overview national dental workforce characteristics and sex-related differences within the profession.

### Data collection

The questionnaire was distributed by email to 189 national dental associations across 133 member countries of the FDI World Dental Federation. The email included a link to the online survey, an information sheet detailing the objectives of the study, and a declaration of consent for data collection. The survey incorporated completeness checks, predefined response options (yes, no, not available, not known), a back-switch option before final submission, and a completion status indicator. Responses were submitted directly through the digital platform. All information was provided voluntarily, anonymously, and free of charge by the participating associations, with country affiliation being the only identifying variable. Data were collected using the electronic research platform Research Electronic Data Capture (REDCap), which was available online from 1 January 2021 to 30 April 2021. All procedures adhered to the ethical standards of the local research commission and the 1964 Declaration of Helsinki, including its subsequent amendments.^23^ According to the Swiss Human Research Act (810.30 HRA), ethics committee approval was not required for the collection and analysis of completely anonymous association data. Data were collected and managed using REDCap electronic data capture tools hosted at the University of Bern (Switzerland).^24^^,^^25^ REDCap (Research Electronic Data Capture) ‘is a secure, web-based software platform designed to support data capture for research studies, providing (1) an intuitive interface for validated data capture; (2) audit trails for tracking data manipulation and export procedures; (3) automated export procedures for seamless data downloads to common statistical packages; and 4) procedures for data integration and interoperability with external sources’ as described previously.^16^

### Data analysis

Data were first exported to Excel (Microsoft Corporation, Redmond, WA, United States of America [USA]) and meticulously checked for accuracy before being imported into STATA 17.0 (StataCorp LLC, College Station, TX, USA) for statistical analysis.^26^ Absolute and relative frequencies were computed for all variables. Group differences in proportions were evaluated using the χ² test, with Fisher’s exact test applied when expected cell counts were less than 5. Post-hoc analyses examined observed vs expected frequencies, percentages, and contributions to the overall χ² statistic. Items measured on Likert scales were analysed accordingly. A multinomial logistic regression model was employed to assess the influence of country, age distribution within dentistry, sex, type of specialization, academic and professional career position, and association-specific female promotions on the outcomes of interest. Data deemed implausible or erroneous were excluded from all analyses. Statistical significance was defined at *P* < .05.

## Results

### Participation and geographic distribution

**Fig. 1:**
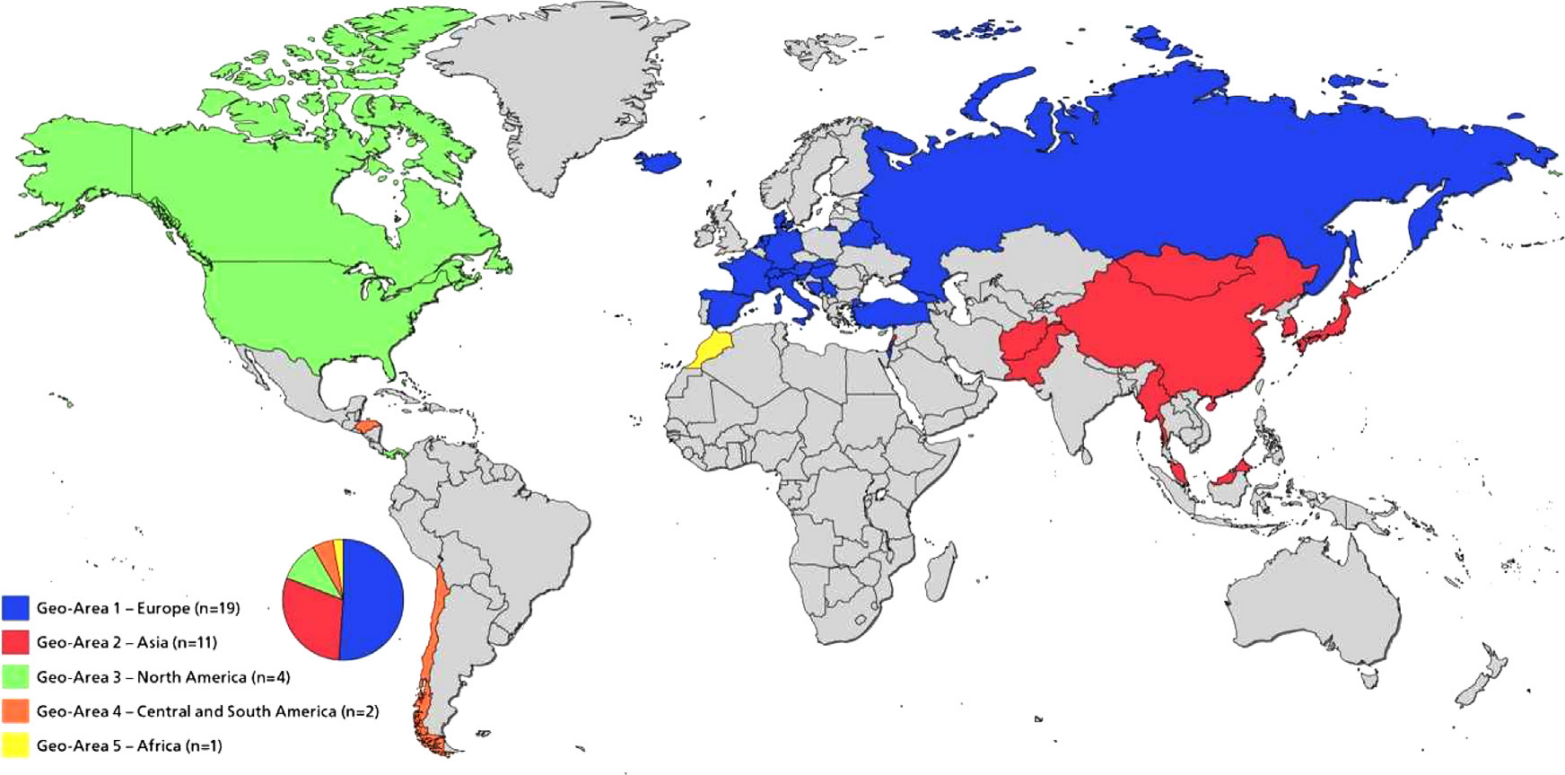
Distribution of participating countries by geographical area.

A total of 44 national dental associations (NDAs) associated with 37 FDI World Dental Federation member countries participated in this survey. Forty-four questionnaires were completed, indicating a response rate of 23.3% (n = 44). All questionnaires were included in the subsequent analyses. These countries/territories participated in the study: Afghanistan, Austria, Bahamas, Belarus, Bosnia and Herzegovina, Canada, Chile, China, Denmark, France, Georgia, Germany, Honduras, Hong Kong, Hungary, Iceland, Israel, Italy, Japan, Lebanon, Malaysia, Mongolia, Morocco, Myanmar, Netherlands, Pakistan, State of Palestine, Panama, Republic of Korea, Russian Federation, Serbia, Slovakia, Slovenia, Spain, Switzerland, Türkiye, and the United States of America. NDAs were categorized into five geographical regions corresponding to continents. The nations of Europe were categorized as geographical area (Geo-area) 1, Asia as Geo-area 2, Central and North America as Geo-area 3, South America as Geo-area 4, and Africa as Geo-area 5 (Figure 1).

### Gender distribution in dentistry and education

Examination of sex distribution revealed significant regional variations. While Geo-area 2 (Asia) reported a 38.1% proportion of female practitioners, Geo-area 3 (Central and North America) had a higher proportion (45.6%). Geo-area 1 (Europe) demonstrated parity, with women constituting 52.1% of the workforce. Geo-area 4 (South America) presented 53.4%, while Geo-area 5 (Africa) emerged as the leader, with women comprising 56.9% of the female dentists. Sex distribution in dentistry varied markedly across age groups. Women comprised 66.7% of practitioners aged 20 to 30 years and 56.5% in the 31 to 40-year cohort. From 41 years onwards, men predominated: 68.2% (41-50 years), 71.4% (51-60 years), 86.4% (61-70 years), and 93.3% (71-80 years). In the 81 to 90-year group, men also represented the majority, while one association reported an equal gender ratio (50:50). According to 80% of the responding countries, the proportion of female dental students exceeded 50%. Conversely, 13.3% indicated a higher proportion of male students, whereas two associations reported an equal sex distribution (non-responders, n = 14). Concerning postgraduate specialization, a significant correlation was observed between the number of female specialists and the total number of dentists (*P* < .001). The linear correlation was weak (r = −0.281) (Table 1). Female specialists accounted for 24.6% in Geo-area 1 (Europe), 15.3% in Geo-area 2 (Asia), and 19.1% Geo-areas 3 to 5 together. In oral surgery, female representation was 2.9% in Geo-area 1 and 1.6% to 1.9% in Geo-areas 2 to 5.

**Table 1:** Correlation between female representation in academic and professional positions and the proportion of female dentists.

Correlation between	r	*P* value
Female leader and percentage female dentists		.399
Female leader and dentist total		.164
Female deans and dentists total		.184
Female professors and dentists total		.293
Female staff and dentists total		.759
Female specialists and dentists total		<.001
Total students and female students	−0.281	.140

Data are expressed as numbers, r indicates the correlation coefficient, and the *P*-value indicates statistical significance. Female deans and presidents were collectively categorized as leaders.

### Women’s representation in academic and professional leadership positions

**Fig. 2:**
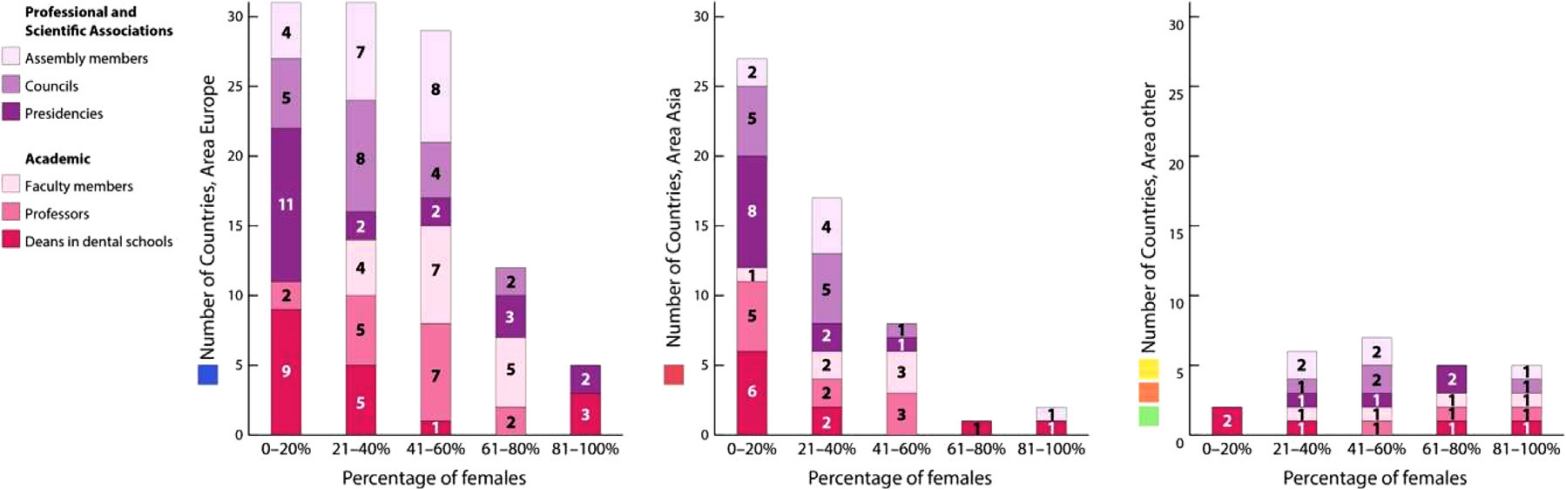
Number of countries reporting female representation (in %) in professional and scientific associations (assembly members, councils, presidencies) as well as academic (faculty members, professors, deans in dental schools) dental leadership positions, stratified by areas.

Across geo-areas 1 to 3 (Europe, Asia, Central & North America), female professorship showed heterogeneous distribution: 34.2% reported 0% to 20% representation, 24.4% reported 21% to 40%, 9.8% reported 41% to 60%, 4.9% reported 61% to 80%, and 26.8% reported 81% to 100%. Regional differences were observed, with geo-area 4 (South America) most frequently reporting 0% to 20% (58.3%), while Geo-area 3 (Central & North America) most often reported 81% to 100% (57.1%) (Figure 2). No significant correlation was observed between the geographical region and the proportion of female professors (*P* = .115). The proportion of female faculty or department heads was predominantly in the 0% to 20% (41.5%) or 21% to 40% range (Figure 2). Geo-areas 3 to 5 (Central & North America, South America, and Africa) were combined for analysis. No significant correlation between geographic region and the proportion of female deans was observed (*P* = .843). No significant association was found between geographic area and the proportion of female staff (χ²(6) = 4.518, *P* = .607). Overall, 22.0% were in the 0% to 20% range and 36.6% in the 81% to 100% range. These findings differed from the distributions observed for female professors and deans. Among respondents, 20.5% reported 0% to 20% female deans in dental schools, 18.2% reported 21% to 40%, and 11.4% reported 81% to 100%. For professorships, 15.9% indicated 0% to 20% and 21% to 40%, while 25% reported 41% to 60%. Female faculty representation differed, with 47.7% reporting 41% to 80% (Figure 2). Concerning scientific associations, 43.2% reported 0% to 20% female presidents, while 11.4% reported 81% to 100%. Female council representation was most frequently reported at 21% to 40% (31.8%), followed by 0% to 20% (22.7%) and 41% to 60% (15.9%). Female assembly representation showed a similar pattern: 21% to 40% (29.5%), 41% to 60% (22.7%), and 0% to 20% (13.6%) (Figure 2). Among the 44 participating associations, 66.7% reported that dentist membership was voluntary. Dedicated female branches existed in 25% (n = 11) of associations, and 22.7% (n = 10) had implemented measures to promote women’s advancement in dentistry. Several countries reported specific measures to promote female dentists (Table 2). In China, the Chinese Stomatological Association (CSA) nominates candidates each year for the 'Chinese Young Female Scientist Project’ and the ‘Future Female Scientist Project’. In Georgia, there are comprehensive structural regulations, including an organized form of paid leave, clear maternity leaves regulations, job security, and full salary continuation throughout the entire period, supplemented by lifelong continuing education and training opportunities. In Germany, the Committee of the German Dental Association (BZÄK) supports young dentists, their families, and practice management through special training courses at the German Dentists' Day, regular surveys among students, and information materials for pregnant dentists in cooperation with the Dentista Association. In addition, a special curriculum in oral surgery for women is offered at the academy in Karlsruhe. In Hong Kong, a Women Dentists Committee (WDC) has been established to bring together the concerns of female dentists and organize relevant activities, including health and community offerings such as yoga classes. The WDC represents female dentists in projects and actively contributes their perspectives. In Hungary, a separate association of female dentists has been founded, which has already held several online meetings. In Italy, workshops and webinars are organized to promote gender equality. There are also projects against gender-based violence, such as ‘Dentista Sentinella’, which trains dentists to recognize signs of domestic violence in female patients. In Japan, the Japan Dental Association (JDA) collaborated with the Cabinet Office on a national survey on women's political participation. In addition, the JDA operates special support pages on its website on reintegration, employment, and different working models for female dentists. In Slovenia, there is an independent association called ‘Women in Dentistry’ that offers continuing education courses specifically for female dentists. In Türkiye, symposia, forums, press releases, and other activities are held to increase the proportion of women in professional representation and leadership. In the USA, programs such as the ADA (American Dental Association) Institute for Diversity in Leadership offer targeted support and leadership training for female dentists and other underrepresented groups.

**Table 2:** Measures and structures in the field of women dentists reported by national dental organizations.

Category	NDA reports
**No sex-specific measures**	Several NDAs stated that they did not implement any specific measures for a particular sex/gender and emphasized a general approach to equal treatment.
**Workshops, webinars, and training courses**	NDAs reported on workshops, webinars, special training courses at conferences, online meetings, and courses for female dentists.
**Formal structures and organizations**	Some NDAs described the establishment of committees, departments, or independent organizations focusing on female dentists or sex/gender issues.
**Targeted projects**	Targeted projects were mentioned, including initiatives to prevent sex/gender-based violence and training courses on recognizing domestic violence.
**Work and support measures**	NDAs reported on regulations regarding paid maternity leave, job security, information services for pregnant female dentists, and support for lifelong learning.
**Surveys**	Several NDAs referred to ongoing or completed surveys on professional or social issues relating to female dentists.
**Information and online services**	Online platforms with information on employment, returning to work, working models, and legal frameworks were mentioned.
**Conferences and public relations**	NDAs reported on conferences, symposia, forums, press work, and other activities with a thematic focus on sex/gender issues.
**Restrictions**	Individual NDAs stated that security-related or regional restrictions limited the implementation of surveys or training opportunities.

### Gender pay gap and equality measures

Regarding gender-related pay, 67.6% of associations reported no disparities, 29.7% indicated data were unavailable or undetermined, and one association (2.7%) reported a 35% pay gap in the private sector.

## Discussion

### Participation and geographic distribution

This international survey encompassed 44 dental associations across 37 FDI World Dental Federation member spanning all continents. It provides a comprehensive overview of gender representation within the dental profession. The findings highlight notable regional disparities and underscore the persistent underrepresentation of women in leadership and academic roles. Analysis revealed significant regional variation in the proportion of female practitioners. Africa (Geo-area 5) had the highest representation (56.9%), followed by South America (Geo-area 4, 53.4%), Europe (Geo-area 1, 52.1%), and North/Central America (Geo-area 3, 45.6%). Asia (Geo-area 2) reported the lowest proportion at 38.1%. These disparities likely reflect underlying differences in cultural norms, healthcare infrastructure, and access to dental education (as highlighted in global workforce trends).^16^

### Sex distribution in dentistry and education

A distinct generational trend emerged: women comprised 66.7% of dentists aged 20 to 30, while in the 71 to 80 age group, male practitioners dominated at 93.3%. This survey indicates a gender shift with increasing age among dental practitioners. This pattern aligns with global observations – women make up between 48% and 75% of the dental workforce, and graduate proportions in several regions (North America, Europe) are near parity, though gaps remain in Asia and Africa.^16^ In this survey, 80% of responding countries reported that female dental students exceeded 50% of the cohort, and in some cases, this reached up to 90%. This trend aligns with findings from the United States, where women represent a significant portion of dental students but are underrepresented in senior academic positions. Factors contributing to this decline may include career interruptions for family responsibilities, limited mentorship opportunities, and systemic biases in career advancement.^6^ Postgraduate specialization also reflected uneven gender distribution: female specialists represented 24.6% in Europe, 15.3% in Asia, and 19.1% in Central & North America. Female dentists who seek to pursue postgraduate specialization predominantly select the fields of orthodontics or paediatric dentistry. Notably, female dentists are underrepresented in the field of oral surgery. Oral surgery seems to remain a typical male specialization. In Europe, women comprise only 2.9% of the professional cohort. Similarly, in Asia, only 1.6% of women choose oral surgery as a specialisation. Evaluating the influence of the rising number of female dentists – including those who engage in part-time work – on the dental care system's ability to fulfil service demands is a complex task.^27^ One study indicated that the probability of dentists engaging in part-time work increases with the number of children. Specifically, dentists with two children were 1.5 times more likely to work part-time, while those with three or more children were nearly twice as likely to do so compared to dentists without children.^27^

### Women’s representation in academic and professional leadership positions

Despite growing female participation in dentistry, leadership roles continue to show significant disparities. Female deans and professors were predominantly reported in the 0% to 40% ranges, with no significant regional differences (*P* = .843). This suggests the persistence of a significant ceiling effect in academia. Scientific associations similarly exhibited limited female leadership, with 43.2% reporting only 0% to 20% female presidents, and only 22.7% implementing specific programs to advance women. Consistent with U.S. data, representation of women in dental academia is growing but remains largely in junior ranks: women held 32.9% of academic faculty positions in 2011 to 2012, increasing to 43.8% by 2022 to 2023, yet remained underrepresented in senior roles with prevalence ratios of 0.6 to 0.8 compared to men.^5^ In 2021, only 18% of U.S. dental schools had female deans, and women held just 18% to 26% of full professorships.^28^ In addition, complex workplace barriers persist – women in dental academia reported significantly more experiences of discrimination, microaggression, bullying, and lack of mentorship compared to their male counterparts.^29^ Intuitively, ‘motherhood’ interrupts career advancement. Bertrand et al.[30] conducted a survey on MBA holders from 1990 to 2006. It has been observed that male and female MBAs initially had nearly identical earnings, male earnings soon surpassed female earnings by almost 60 log points a decade after MBA completion. This gap was largely due to higher career discontinuity and shorter work hours for female MBAs, mainly linked to motherhood.^30^ Finally, existing directors’ behavioural habits, such as seeking people similar to themselves in the selection process for new members, might also explain the persistence of low gender diversity.^31^ Although challenging, introducing quotas is a crucial last-resort strategy to disrupt established networks of deans and academic vice presidents and mitigate existing gender disparity.^32^

### Gender pay gap and equality measures

International data highlight persistent gender pay gaps. While 67.4% of associations reported no disparities, 30.2% indicated that data were unavailable or undetermined, and one association reported a 35% pay gap in the private sector. Contributing factors may include differences in work hours, practice ownership, and specialty choices, as well as systemic undervaluation of women's work. In the UK, NHS data show that female dentists earned approximately 33% to 34% less taxable income than male dentists in 2020/21 and 2021/22.^33^ Similarly, a UK industry report found a gender pay gap of around 39.3%, despite women constituting over 55% of the dental workforce.^34^ US studies suggest that male dentists earn substantially more than female dentists, with some analyses indicating a 22% difference after adjusting for personal, employment, and household factors.^6^ Only about 27% of this wage disparity is explained by these observable factors, leaving most of the gap unexplained and likely structural¹. Earlier research found that 62% to 66% of the gender pay gap among dentists could not be accounted for even after multivariate adjustments.^35^ A 2017 ADA Health Policy Institute report suggested that male dental providers earned up to 54% more than women, and even after controlling for age and hours worked, a 36% unexplained gap persisted.^35^ Against this backdrop, it seems remarkable that the NDAs in this study – except for a single response from the private sector in one country – either reported no inequalities or stated that they did not have any relevant data on this issue. These disparities are further compounded by limited implementation of equality measures: only 22.7% of dental associations had programs aimed specifically at promoting women in leadership or scientific roles.^6^ This study indicates the limited availability of mentoring programs for female dentists. Future research should focus on identifying best practices to enhance and expand these mentoring initiatives.^36^ Beyond leadership and income disparities, gender inequalities in dentistry might also have an impact on the reality of care and access to oral health. Dutch dental practices often provide insufficient information on their websites about the treatment of frail older patients.^37^ This points to structural deficits in communication, accessibility, and practice adaptation to an aging population. Since older women make up the largest share of the very old population, such information gaps could exacerbate existing inequalities in care, especially for vulnerable patients. At the structural level, the phenomenon of ‘provided by women, led by men’ continues to shape the health sector: although women make up around 70% of global health and social care professions, they are significantly underrepresented in leadership and decision-making positions.^38^ Systemic inequalities in status, remuneration, and career development not only influence individual careers, but might also potentially influence strategic priorities within health systems. In addition, gender-specific differences become apparent early on in the education process. Significant differences were reported in the career motivations of first-year dental students, suggesting that gender-specific expectations take effect at an early stage.^39^ Previous research has also shown differences in oral health behaviour and the use of dental services between men and women.^40^ Overall, these findings illustrate that gender inequality in dentistry is not solely a question of representation in leadership positions, but has far-reaching implications for workforce development, care planning, and gender-sensitive, equitable oral health care.

## Policies of global associations

The findings on female representation in academic and professional leadership structures in dentistry are consistent with key international strategic frameworks. In its Global Strategy on Human Resources for Health: Workforce 2030, the World Health Organization emphasizes that despite the increasing number of women in the health sector, structural inequalities in leadership positions and remuneration persist and impair the performance of health systems.^41^ The targeted promotion of women in leadership roles is defined as an essential prerequisite for sustainable health development. The FDI World Dental Federation also highlights the importance of a resilient and diverse dental workforce in ‘Vision 2030 – Delivering Optimal Oral Health for All’.^42^ Against the backdrop of the increasing feminization of the profession and simultaneous underrepresentation in leadership positions, our data underscores the relevance of inclusive governance and career structures. Furthermore, the findings correspond with the United Nations' Sustainable Development Goal 5 (Gender Equality).^43^ UNICEF emphasizes that gender equality is a measurable structural determinant of long-term health and workforce development.^44^ Overall, our results position the promotion of female leadership in dentistry as a strategic contribution to global goals of sustainable and equitable health care.

## Limitations and future directions

When interpreting the results of this survey, a few limitations should be kept in mind. This survey is constrained by a small sample size, absence of comparison groups, and inability to establish causality. A key limitation of this study is the response rate, which may be associated with potential sampling and non-response bias. This reflects structural challenges in collecting gender-specific workforce and leadership data, as such information is not systematically recorded in many countries or has been given lower institutional priority to date. Several organizations were therefore unable to provide reliable data. Fifty per cent of the participating associations were based in Europe, reducing the world-wide analysis. The geographical overrepresentation of European NDAs probably reflects regional differences in the degree of organization and establishment of female dental networks and may have limited the global generalizability of the results. Another limitation of the study is the lack of reliable data on gender-specific income differences. The questionnaire was administered exclusively in English and lacked validation across other languages which may have affected the responses. One potential factor contributing to the low response rate to the survey may have been the global COVID-19 pandemic, which resulted in a decreased prioritization of data entry by the associations, contingent upon the prevailing circumstances. Additionally, data accuracy could not be independently confirmed. To promote gender equality in academic dentistry and medicine, several strategies should be implemented. Leadership positions should accommodate job-sharing arrangements, as dual leadership is common in many sectors but remains rare in university medicine. Appointment committees should achieve gender parity and include university lecturers, with equal opportunity officers actively involved in selection decisions. Institutions should establish women-friendly structures, offering childcare and streamlined meetings. Future improvements should be undertaken implementing family-friendly work structures, such as flexible hours and childcare support. This seems to be important to create gender equality. Furthermore, flexible learning opportunities are part of the process. Online learning and online postgraduate education play a crucial role in promoting gender equality in dentistry, even in the post-COVID era.^45^ Current female leaders should focus on empowering the next generation, serving as mentors^46^ and creating tangible opportunities for other women to advance into leadership roles. Support both from female and current male leaders is required to change these metrics. Understanding women's current roles and history in academic and political medicine is key to improving future leadership opportunities.^47^ According to a US study, the three most important benefits of mentoring are better overall professional development, the development of a career plan and an expanded professional network.^48^ Additionally, the establishment of databases to monitor women’s career progression and leadership advancement could be helpful. Overall, removing structural and cultural obstacles that hinder women’s rise to top positions is crucial to achieving parity at all levels.

## Conclusions

This global survey emphasizes persistent gender disparities in dentistry world-wide, particularly in professional leadership and academic positions, despite the growing proportion of women in the profession. Structural and societal barriers hinder career advancement, the lack of reported data on gender-related pay differences highlights the need for further research to support transparent remuneration policies. Promotion of women into leadership roles, and the creation of inclusive work environments that support work-life balance are required. Mentoring programs and gender equality initiatives are essential. Further research is needed to understand the underlying causes of these disparities and to develop effective strategies to achieve gender equity in dentistry.

## Conflict of interest

None disclosed.

## Appendix Supplementary materials

Image, application 1
